# Experiences of family caregivers of persons living with mental illness: A meta-synthesis

**DOI:** 10.4102/curationis.v42i1.1900

**Published:** 2019-09-19

**Authors:** Esther I. Ntsayagae, Marie Poggenpoel, Chris Myburgh

**Affiliations:** 1 Department of Nursing Science, University of Johannesburg, Johannesburg, South Africa; 2 Department of Educational Psychology, University of Johannesburg, Johannesburg, South Africa

**Keywords:** caregiving, coping, experiences, family caregivers, mentally ill patients, meta-synthesis

## Abstract

**Background:**

Meta-synthesis is used to generate and understand new insights from a qualitative perspective. Caregiving is associated with a range of physical and psychological symptoms. Caregivers bear the brunt of caregiving and this has become worse since the inception of de-institutionalisation, as more patients are discharged into the community under the care of their families.

**Objectives:**

The purpose of this study was to synthesise phenomenological qualitative studies and create a comprehensive chronicle of phenomena of family caregivers’ experiences of caring for relatives living with mental illness.

**Method:**

Google Scholar and different electronic databases, which included CINAHL, MEDLINE, EBSCO and PubMed, were searched using keywords for relevant studies published from 1994 to 2014. To obtain an in-depth view of caregivers’ lived experiences, a qualitative meta-synthesis was employed to review the findings of 10 studies.

**Results:**

A total of 10 studies were included in the meta-synthesis. The family caregivers described their caregiving experiences under four themes: perceived responsibility of caregiving, experiences of emotional effect, experiences of support needs and experiences of changed perspective.

**Conclusion:**

The meta-synthesis revealed a lack of emotional coping among the family caregivers. This calls for robust family caregiver interventions to facilitate their mental health.

## Introduction

Mental health disorders are a major health issue for the global community, constituting five of the 10 leading causes of health disability in the world (WHO 2001). Botswana saw a moderate rise of 5% from 39 778 psychiatric attendances in 2007 to 41 908 in 2009 (Health Statistics Report 2007). The rising number is tantamount to more people being cared for in the home setting.

The shift in mental health treatment philosophy from institutions to their homes had a profound, but rarely acknowledged, effect on the family (Seloilwe & Thupayagale-Tshweneagae 2007). Mental illness is a chronic illness and it necessitates family caregiving throughout the lifespan of the relative living with mental illness. Hence, the family caregivers shoulder a large share of the long-term responsibilities. Research suggests that mental illness is a devastating illness for both the caregiver and the patient (Moahi 2007; Seloilwe 2006). Caregiving affects the family in various ways. Caring for relatives living with mental illness affects the physical, psychological and socio-economic well-being of the caregivers, as well as their capacity to cope with and adjust to those circumstances. Without support, they end up compromising their own health and well-being (Jack-Ide, Uys & Middleton 2013).

Caregivers for relatives living with mental illness face different challenges from other caregivers of long-term conditions, in that they sometimes fear for their lives as their relative may become aggressive (Pusey-Murray & Miller 2013). Caregiving may also strain family relations and other coping resources, which can be felt more acutely in situations where community rehabilitation resources are lacking. Various other experiences are also reported in the literature; Jönsson et al. (2011) found that families had to contend with feelings of worry and powerlessness to maintain normality in their lives.

The focus on this study, therefore, is on understanding the experience of caregiving from the perspective of family caregivers providing care to relatives living with mental illness. The unfavourable impact of caregiving on family caregivers’ health has been reported as requiring attention (Van Wijngaarden, Schene & Koeter 2013). Bhatia and Gupta (2003) contended that it is therefore imperative to provide unrelenting support to family caregivers and individual members to enable them to re-focus and learn to manage illness-related roles and tasks. Following de-institutionalisation, and rapid demographic and socio-economic changes because of urbanisation (Balogi 2004; Kimani & Kombo 2010), it is timely or long overdue to consider what is done about caregivers’ mental health as an essential component of one’s overall health.

### Problem statement

Although enormous progress has been made in studying and documenting the lived experiences of family members caring for relatives living with mental illness in Africa (Jack-Ide et al. 2013), to date there has not been any integration of available literature and there is not much information about family caregivers’ support. There is thus a need to merge these findings to understand the complexity of caregiving more than looking at isolated pieces of information, which in many instances lack contextual relevance.

Consequently, the study seeks to present a meta-synthesis of phenomenological studies conducted on family caregivers’ lived experiences of caring for relatives living with mental illness in sub-Saharan Africa, and to create a comprehensive chronicle of phenomena in the findings of studies conducted.

### Purpose

The purpose of the research was to meta-synthesise phenomenological studies conducted on family caregivers’ lived experiences of caring for relatives living with mental illness.

## Research design and method

The meta-synthesis was based on Walsh and Downe’s (2005) method, as well as the strategy by Noblit and Hare (1988).

### Population and sampling

The population consisted of phenomenological studies that generated experiential descriptions about caring for relatives living with mental illness. Bondas and Hall (2007) recommend that at least 10–12 studies should be purposively included in the meta-synthesis to create a meaningful and valid meta-synthesis. A total of 10 purposively selected phenomenological studies were included for the review.

Inclusion criteria were studies that: (1) were on family caregivers who have experienced caring for a mentally ill relative in the home environment, (2) explored experiences of family who have experienced caring or caregiving from the perspective of a family member, (3) were on families who take care of mentally ill relatives and (4) explored qualitative data (phenomenological research) which is published in English.

Studies were excluded if they (1) were review papers and abstracts, or if they (2) were meta-synthesis studies on caregivers’ experiences.

### Data collection

A systematic search of qualitative research studies was undertaken with the assistance of the university librarian. Relevant literature was identified through Google Scholar and different electronic databases that included the Web of science, EBSCO host, PubMed and MEDLINE from 1994 to 2014. The search terms used were *caregiver* or *family caregiver* AND *mental illness* or *mentally ill* or *persons living with mental illness* AND *caregiver burden or strain or coping* AND *sub-Saharan Africa*. A coding form that was utilised for data collection consisted of objectives, sampling, research design, research methods, research results and recommendations.

**FIGURE 1:**
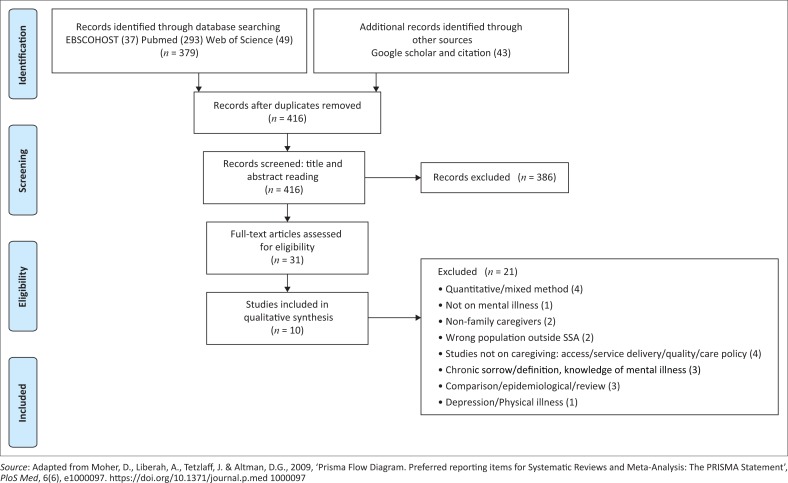
Prisma flowchart – study selection process.

A four-step process for the meta-synthesis based on Walsh and Downe’s (2005) method, as well as the strategy by Noblit and Hare (1988), was adopted. The Prisma flow chart (Figure 1) helps to illustrate the process.

#### Selection and appraisal of relevant research studies

For this meta-synthesis, the researcher consulted the university librarian to locate and select research studies. Screening was based on titles and abstracts, and eligibility was based on the methodology, trustworthiness, data analysis and results. The search strategy comprised a search to identify relevant keywords contained in the title, abstract and subject descriptors. However, no studies were rejected on the basis of quality.

#### Relevance and selection of studies

In this step, an evaluation of the relevance and finalisation of the studies was done. In ensuring transparency, the researcher reported on the process of selection. Cross-checking was done for all the selected studies. Walsh and Downe’s (2005) guidelines were used to determine the relevance of the study by comparing various study results.

### Trustworthiness

Walsh and Downe (2005) stated that there were no widely agreed upon criteria to assess the quality of qualitative studies. However, the Critical Appraisal Skills Programme (CASP 2018) published a checklist that mentions three broad issues that are to be considered when appraising a systematic review study. An important criterion for selection is adhering to the standpoint or research focus. Therefore, in ensuring trustworthiness, the criteria of clarity, structure, coherence, scope, generalisability and pragmatic utility (Bondas & Hall 2007), and Walsh and Downe’s (2005) methodological rigour were used to appraise the studies. This was done in the form of a coding scheme. Each stage of the research process was assessed for clarity, congruence, transparency and methodological underpinnings. A checklist was then developed to rate the quality. No studies that met the inclusion criteria were excluded on the basis of quality. The quality ratings ranged from 6.5 to 10.

The synthesis and results step involved reviewing studies, identifying and summarising key themes. These were verified against each other, resulting in findings that were synthesised into new concepts. Following the identification of relevant studies, a comparison and contrast exercise of the key concepts in the results was done. The authors’ understanding of concepts and relations were identified in each study. Summarisation was tabulated to present inferred themes and concepts from the narrative accounts in caregiving. Thematic synthesis involved line-by-line coding of the findings of the primary studies and the development of descriptive and analytic themes. The classic method of Noblit and Hare (1988) was followed.

### Ethical considerations

Ethical clearance was obtained from the university’s Faculty of Health Science Research Ethics Committee (REC-01-180-2015). Human subjects were not the objects of the synthesis research. Therefore, the study posed no risks to participants. Nevertheless, the ethical processes adhered to focused on accuracy and honesty in reporting the findings on the research done. The researcher did not deliberately misinterpret facts about the studies, remained objective and refrained from making value judgements with regard to the conducted studies.

## Findings

### Description of selected studies

The meta-synthesis sample was composed of 10 research studies. Six of the studies were research theses (five master’s and one doctoral thesis), and four were research articles from various countries in sub-Saharan Africa and from different academic disciplines, including nursing and health studies. The studies aimed to explore experiences of family members caring for, living with, or having a mentally ill relative, except for one study that focused specifically on schizophrenia. The studies identified were undertaken in Botswana, South Africa, Namibia, Swaziland, Tanzania and Kenya, from various disciplines. Research results from 102 caregivers’ experiences are presented. Data were collected through in-depth interviews, focus group discussions and naive sketches. Participants were grandparents, parents, siblings, spouses, nephew and nieces. Participants’ ages ranged from 17 to 79 years. Caregiving experiences ranged from less than 2 years to more than 20 years. Female participants took the lead in the reviewed studies (refer to Table 1).

**TABLE 1:** Characteristics of reviewed studies included in the meta-synthesis.

Author	Country	Study title/topic	Data collection method	Sample size	Data analysis	Design
Banyini (2012) Study 1	South Africa	Experiences of family members caring for a long-term mentally ill patient at Letaba, Limpopo Province	In-depth interviews, direct observations and field notes	4 members in 1 family	Open coding	Qualitative, exploratory, descriptive
Moahi (2007) Study 2	Botswana	Experiences of family members living with a family member suffering from chronic schizophrenia in Lobatse	In-depth individual interviews, naive sketches and observational notes	12	Open coding	Qualitative, exploratory, descriptive, contextual
Ngqoboka (1998) Study 3	South Africa	The family’s experiences of having a mentally ill family member	In-depth semi-structured interviews, field notes and observational notes	5	Open coding	Qualitative, exploratory, descriptive, contextual
Zwane (2012) Study 4	Swaziland	Family caregivers’ lived experiences of violence at the hands of their mentally ill relatives in Swaziland	In-depth semi-structured phenomenological interviews and field notes	6	Open coding	Qualitative, exploratory, descriptive, contextual
Mphelane (2006) Study 5	South Africa	The role played by the families in support of their mentally ill relative in a rural community in Limpopo Province	In-depth semi-structured phenomenological interviews and field notes	8	Open coding	Qualitative, descriptive, contextual, exploratory
Shifiona (2014) Study 6	Namibia	Facilitating the mental health of individuals living with chronic mental illness in the North West Health Directorate – Northern Namibia: a community involvement approach	In-depth phenomenological interviews, focus group discussions and field notes	4 case studies making 8 family members	Tesch’s open coding	Qualitative, descriptive, contextual and theory generative research design
Ambikile and Outwater (2012) Study 7	Tanzania	Challenges of caring for children with mental disorders: Experiences and views of caregivers attending the outpatient clinic at Muhimbili National Hospital, Dar-es-salam, Tanzania	In-depth interviews, focus group discussion and field notes	8	Content analysis	Qualitative, descriptive
Monyaluoe et al. (2014) Study 8	RSA	Experiences of families with a mentally ill family member	In-depth interviews	14	Open coding	Exploratory, descriptive, contextual, qualitative
Seloilwe (2006) Study 9	Botswana	Experiences and demands of families with mentally ill people at home in Botswana	In-depth interviews, focus group discussion and field observations	30	Open coding and axial coding	Exploratory, descriptive, contextual, qualitative
Wankiiri, Drake and Meyer (2013) Study 10	Kenya	The lived experience of families with a mentally ill family member	Interviews	7		Qualitative, descriptive, phenomenological

*Source*: Ntsayagae, E.I., 2017, ‘A psycho-educational programme for caregivers of mentally ill persons to facilitate their mental health’, pp. 82–87, Thesis: D Cur Psychiatric Nursing, University of Johannesburg, Johannesburg

### Findings of the interviews: Family caregivers’ experiences for relatives living with mental illness

A line of argument of 10 studies yielded four overarching themes: (1) perceived responsibility of caregiving, (2) experienced emotional effect, (3) experienced support needs and (4) experienced changed perspectives (see Table 2). The four themes are presented in this article with direct quotes from the included studies.

**TABLE 2:** Family caregivers’ experiences for persons living with mental illness.

Themes	Sub-themes
Theme 1: Family caregivers experienced perceived responsibility for caregiving	Family caregivers experienced provision of care Family caregivers experienced role shift Family caregivers experienced altered responsibilities
Theme 2: Family caregivers experienced emotional effect of caregiving	Family caregivers experienced feelings of hopelessness and helplessness Family caregivers experienced feelings of shame and fear Family caregivers experienced feeling of powerlessness
Theme 3: Family caregivers experienced support needs	Family caregivers experienced inadequate informational support Family caregivers experienced ineffective coping Family caregivers experienced societal challenges
Theme 4: Family caregivers experienced changed perspective	Family caregivers experienced effective coping mechanisms Family caregivers’ positive experiences

*Source*: Adapted from Ntsayagae, E.I., 2017, ‘A psycho-educational programme for caregivers of mentally ill persons to facilitate their mental health’, p. 110, Thesis: D Cur Psychiatric Nursing, University of Johannesburg, Johannesburg

#### Theme 1: Family caregivers experienced perceived responsibility of caregiving

Family members remain the primary caregivers for relatives living with mental illness. The caregiving responsibility was seen in all stages of illness. The family caregivers took care of the day-to-day needs of the relative with mental illness, which included providing meals, assisting in activities of daily living, monitoring their treatment and their mental state, observing for signs of relapse and helping their relatives to access services. Family caregivers stated that they are responsible for the day-to-day care of their relative, and this often leads to role shifts and altered responsibility.

There is no problem that is bigger than caring for the mentally sick brother, because he is forever…I don’t know when it will end completely. (Wankiiri, Drake & Meyer 2013:60)

I rely on God and mental hospital for care of my relative. (Moahi 2007:50)

**Family caregivers experienced the provision of care:** Family caregivers extended practical support by proving care to meet the needs of their relative living with mental illness. A great deal of time and effort was spent in providing care. Caring required numerous sacrifices with regard to social and occupational life. This also included financial support in terms of travel expenses, funding treatment and providing food. The family caregivers also expressed difficulties in maintaining their relative, in terms of providing for their relative’s needs.

When he is well he eats too much, but other times when he is given food he just throws it away. (Banyini 2012:78)

Family caregivers experienced physical exhaustion because of taking care of their mentally ill relative (Ngqoboka 1998). Through providing around the clock care, family caregivers can become victims of physical and emotional exhaustion.

It is reported that family caregivers also encountered frustration when their mentally ill relative refused medication and food.

The support that we give him is to ensure that he has taken his medication correctly because he often refuses to take his medication. I always beg him. (Mphelane 2006:30)

To me the transport fare to collect free medication is almost equivalent to buying the drug …this has caused me a lot of financial problems because I come from very far to come to the hospital and I have to do this every month. (Wankiiri et al. 2013:60)

**Family caregivers experienced role shift:** The nature of mental illness affects the family members in various ways. Caregiving can challenge long-standing family roles. Everything the family does is planned around the relative with mental illness. The caregiver role exposes family members to new experiences and ranges of emotions. Taking care of a mentally ill relative can interfere with a person’s ability to execute daily chores, and taking care of the self and other family members. Family caregivers reported that there was a role shift, as they often ended up altering their responsibilities and had to combine caregiving with attending to their everyday roles.

One family caregiver said:

He used to take care of himself, but now you have to tell him to do everything, like washing clothes and asking him to take medicine. (Wankiiri et al. 2013:60)

The family caregivers’ lifestyle had to change to accommodate the caregiving role. Similar findings were reported by Mavundla, Toth and Mphelane (2009); families adjust their social lives to the needs of their relatives living with mental illness, which is a source of stress as they no longer have time for themselves. Family relationships can be weakened because of the caregiving conflict among family members. Role shifts can affect the family dynamics as it sometimes contributes to disagreements among family members.

**Family caregivers experienced altered priorities:** Another common thread among the studies was that there were altered priorities as care was centred on the mentally ill relative. Mental illness involves impairment in one or more important areas of functioning (Frisch & Frisch 2011). There is a need for ongoing adjustments in families regarding the constant presentation of changes in mental illness. Some lifestyle changes are necessary to accommodate the caregiving role. The chronic nature of mental illness and the ongoing demand to be available to take care of a mentally ill relative were some of the difficulties reported by family caregivers. They expressed concern about their altered priorities, particularly when they had to provide for their relative living with mental illness.

It is very difficult because I am not working, and I am self-employed and whatever I put on the table I share with him. (Monyaluoe et al. 2014:4)

Family caregivers invest a lot of energy into the caregiving role and tend to put anything that affects them on hold. One family caregiver said:

The biggest problem that I have is that I am never able to work because I have to look after him, it requires me to be around all the time. (Wankiiri et al. 2013:59)

#### Theme 2: Family caregivers experienced emotional effects

This theme relates to the emotional experiences of caregiving. The theme is further divided into three sub-themes: feeling hopeless and helpless, feelings of shame and fear, and feeling powerless. Family caregivers experienced a variety of negative emotions.

**Family caregivers experienced feelings of hopelessness and helplessness:** Because of the complexity of the nature of the illness, it is not surprising that family caregivers responded to caregiving with emotions of their own. The emotions were varied and might include a need to help the relative, feelings of failure and helplessness as the disease progressed. Most often helplessness and fear went hand-in-hand. The family caregivers felt helpless when they could do nothing about their relative’s condition and when they were fearful of his or her aggressive behaviour. Helplessness was observed when family caregivers could do nothing about the situation. This relates not only to basic knowledge on caregiving, but also to feeling helpless as well.

I rely on God and mental hospital. (Moahi 2007:50)

Family members felt helpless when they could do nothing about their relative’s aggressive behaviour. One family caregiver said:

He destroys household utensils and furniture and throws them to the neighbors’ houses. He also throws stones which can hurt. When he is like that no one approached him, we leave him and he remains alone. (Banyini 2012:79)

Negative emotions experienced included emotional pain, frustration and helplessness.

**Family caregivers experienced feelings of shame and fear:** Most of the family caregivers lived in fear of being stigmatised because of the verbal assaults and outburst of their relative. This affected their mental well-being. One family caregiver said:

I am hurting because of words of rejection and stigma that are uttered by our neighbours about our mentally ill person and us. (Moahi 2007:52)

Stigma and shame against people living with mental illness remain unabated. Anything regarding mental illness has negative publicity and caregivers are challenged twice; on one hand, they have to bear the challenges of having to deal with bizarre behaviour that is often frightening, and on the other hand, they face prejudice because of misconceptions about mental illness (Sadik et al. 2010). One caregiver said:

We felt as if we were alone in an island, there was nobody to turn to, friends and families were at distance, there was nobody visiting us. (Ngqoboka 1998:35)

The consequences of shame and stigmatisation affected access to social roles, leading to social isolation. Caregivers also experienced fear for themselves and for their relative’s safety.

Every time there is fear that he might move away and cause havoc. (Wankiiri et al. 2013:61)

**Family caregivers experienced feelings of powerlessness:** Powerlessness compounds feelings of uncertainty, frustration and fear that the mentally ill relative may become dangerous. In all 10 studies, family caregivers reported feeling powerless. Family caregivers experienced negative thoughts related to self-worth, hope and power. These emotions can affect one’s sense of well-being. They felt powerless when they could not do anything about their relative’s aggressive behaviour. One family caregiver said:

He will dish up without even washed his face and hands. And when I tell him to go and wash first and then you have provoked him. He will shout one word and that will be enough for you to keep quiet and leave him to do what he wants. (Zwane 2012:32)

Family members became powerless because they had insufficient knowledge about chronic mental illness and upon realising that they are dealing with chronic illness. Factors that affect powerlessness among caregivers are complex and multidimensional; they become powerless when they cannot influence the outcome.

Family caregivers also had feelings of frustration, guilt and powerlessness invariably communicated to them, as the relative living with mental illness did not always appreciate the caregiving efforts and sometimes blamed them for their failures. They expressed powerlessness and frustrations as they did not know how their relative would present.

It is very difficult to plan for him because his behaviour changed every time; he does things unexpectedly so you need to watch him closely. (Wankiiri et al. 2013:60)

#### Theme 3: Family caregivers experienced support needs

The theme is divided into inadequate informational support, ineffective coping and societal challenges. Caregiving can be a lonely and isolating experience for family caregivers. Social support is considered to be a protective factor against psychological difficulties. Findings suggest that the stress family caregivers experienced is associated with a lack of essential support.

**Family caregivers experienced inadequate informational support:** Most family caregivers reported inadequate informational support. This is one of the contributing factors to caregiver burden (Brodaty & Donkin 2009). Families often lack the knowledge and skills needed to assist their mentally ill relative (Jones 2010). With inadequate support, family caregivers felt helpless, which led to feelings of discomfort as they were unable to assist the relative with mental illness. Family caregivers reported failing to understand the illness, as well as the behaviours displayed by their relative. On many occasions, they claimed to be unable to assist their relative because they did not understand the illness and thus failed to give continued support (Banyini 2012).

**Family caregivers experienced ineffective coping:** Coping is the process of managing external or internal demands that exceed the resources of the person. The majority of the studies reviewed made reference to ineffective coping. Because of the limited formal support, coping with the demands of caregiving becomes difficult. The family caregivers described caring for their relative living with mental illness as challenging, stressful and painful.

Ineffective coping was twofold. Firstly, family caregivers were in denial and always lived in fear because of their relative’s unpredictable behaviour. Secondly, family caregivers lacked knowledge, and were uncertain about practically caring for their relative with mental illness. This was noted in two articles (Shifiona 2014; Zwane 2012):

It is difficult to have a child with an illness that causes confusion. (Shifiona 2014:115)

Family caregivers were less positive about their caregiving experiences. This was noted among the majority of family caregivers who are elderly people. Many were worried about the future because of a lack of support. Ineffective coping had an impact on their emotional well-being. Because of insufficient knowledge, family caregivers became powerless and were unable to cope with caregiving. One sister said:

There is no problem that is bigger than caring for the mentally sick brother, because he is forever…I don’t know when it will end completely. (Wankiiri et al. 2013:60)

Feelings of powerlessness following an increase in symptom severity led the family caregivers to seek support from healthcare professionals. Ineffective coping sets in when there are feelings of helplessness.

**Family caregivers experienced societal challenges:** The studies portrayed societal challenges. The demanding caregiving role did not only interfere with their day-to-day work, but reduced outside social opportunities as well. Some reported that they were not invited to events because of the unpredictability of their relative’s illness. The family caregivers felt cut off from society as they steadily lost friends and social contacts. This theme was confirmed by results from studies conducted by Wankiiri et al. (2013); the same findings were also reflected by Moahi (2007).

Caregiving has profound effects on the health of the family caregiver caring for a relative living with mental illness because they not only responded to the person’s symptoms, but also negotiated with other difficult family members. Family caregivers ended up having to balance their own needs and the needs of relatives living with mental illness.

Sometimes family caregivers worried about their children’s safety, which could lead to family breakdown as children were sent to live with other relatives for their safety. The family might also be stigmatised. Stigma could arise from a number of factors such as suspicion, lack of knowledge, ignorance, belief systems, fear and the exclusion of people believed to be different (Iseselo, Kajula & Yabya-Malima 2016). This results in a sense of social loss as described by most participants. This is what one caregiver said:

We felt as if we were alone in an island, there was nobody to turn to, friends and family were at a distance no one was visiting us. (Ngqoboka 1998:35)

The mental health benefits of social support were mainly evident during stressful periods. Taylor and Stanton (2007) reported that social support reduces psychological distress and contributes to physical health and survival. The caregivers felt isolated from society because of the disruptions of their social life as they had to care for the patient. Social discrimination was also caused by the stigma attached to mental illness. Stigmatisation and social isolation were a prevalent experience among the family caregivers who had to contend with social relationships that were strained by lack of knowledge and negative attitudes about mental illness (Shamsaei et al. 2013). Some statements by caregivers were depictive of loneliness. One caregiver said:

[*W*]hen he relapses you cannot call in the neighbors all the time for help… you suffer alone and at the same time you also feel for the child. (Shifiona 2014:115)

#### Theme 4: Family caregivers experienced changed perspective

With adequate support, family caregiving can be a rewarding experience. Family caregiving responsibility affected the way family caregivers perceived life; they had to learn how to cope with the illness and to find a balance in the provision of care. These perspectives were mediated by emotional sequelae, feelings of being overburdened and unmet support needs from their family and healthcare professionals.

**Family caregivers experienced effective coping mechanisms:** Coping is the process by which an individual contends with or deals with a situation to alleviate, relieve or remove stress (Uys & Middleton 2014). Coping enables the individual to remain unaware of an unpleasant reality as if it did not exist. Some caregivers used denial and blame. An inability to cope has been shown to present in physical symptoms like sleeplessness, unrest and anger. This can have a negative impact on the caregivers’ mental health. According to Wilkinson and Lynn (2005:122–130), the course of the relative’s illness shapes the caregiver’s experience.

The family caregivers used different coping mechanisms: some positive and others negative. To decrease stress and thereby maintain mental health, family caregivers reported adjusting and adapting to the situation. By learning to accept the situation and by implementing alternative coping methods, family caregivers’ mental health can be improved. Not accepting the behaviour of a mentally ill relative may cause additional stress not only to the family caregiver but also to the relative as well (Kung 2016). Some family caregivers said they made efforts to accommodate and accept their relative living with mental illness. They also avoided conflict and upsetting their relative by tolerating their behaviour. However, to cope with their feelings, others tended to be in denial and blame God for the illness. One said:

I even asked God to say: What have we done? (Monyaluoe et al. 2014:5)

This is supported by Endrawes, O’Brien and Wilkes (2007), who noted that family caregivers sometimes tend to look for someone or something to blame.

Families’ views linked mental illness to spirituality as some form of coping. Ngqoboka (1998) noted that families could have psychological defence mechanisms to cope with stressful situations. A belief in God and hope for the future was identified as such a coping mechanism. Also, despite negative feelings, family caregivers had hope that their relatives would be cured if they were kept in hospital:

I hoped my brother will be cured if he stayed in hospital. He would be discharged on treatment so that he continues taking it. He is not so bad. (Ngqoboka 1998:37)

Some have learnt to accept the situation because they felt they had no other alternative. One family caregiver said:

Really I have accepted it because I have already been given, but it’s a big task, it’s a very big task. (Ambikile & Outwater 2012:5)

One other way of coping that was reported was being optimistic and being hopeful that God would heal the mentally ill relative. This was supported by Endrawes et al. (2007), where caregivers learnt to cope better by being hopeful that God would heal the relative living with mental illness.

**Family caregivers’ positive experiences:** The family caregivers’ experiences were not unanimously bleak. Some family caregivers experienced satisfaction and had positive feelings about caregiving, especially after empowerment and receiving interventions from the hospital. They appreciated the interventions they received from the hospital. Upon securing treatment for the affected individual, family caregivers reported relief and hope.

I thank the nurses for their support and visits. (Moahi 2007:65)

I wish the health workers could continue visiting us and empower us and see that we are meeting the demands of caring for the sick. (Moahi 2007:65)

Seloilwe (2006) stated that family caregiving also brought the family together. Someone responsible would be accountable for providing care. Other family members would assist in providing money, food or materials necessary for providing care.

We had a way to decide who was going to take care of my mother… and even pool our efforts to provide her with a salary she was earning. (Seloilwe 2006:264)

## Discussion of findings

The findings provided insight into the caregiving experiences of family caregivers for relatives living with mental illness. The important theme was that the family caregivers experienced challenges with caregiving; however, they also experienced changed perspectives regarding caregiving. Within the theme of caregiver responsibility, the family caregivers felt overwhelmed and were frustrated. They did not know how to seek help. It has also been reported by various studies that caregiver responsibility may lead to a physical and emotional burden for the caregivers (Schulz & Sherwood 2008; Shah, Wadoo & Latoo 2010).

The caregiving impact has been endorsed by Trondsen (2012), who noted that mental illness strained relationships and affected physical and mental well-being of caregivers. In contrast to the majority of studies that focused on helping the caregivers to cope with assisting their relative to adhere to treatment, this review brought to light the effects of caregiving on mental health.

A key factor influencing fear was helplessness. Helplessness seems to be a determinant of fear for most family caregivers. However, fear also emanated from being stigmatised and fearing for their relative’s safety. When they did not know what to do, knowledge could have helped reduce their fear. One clear implication is that helplessness affects fear through a lack of knowledge of what to do. It is significant that the experiences of family caregivers seemed to be influenced by support and knowledge.

The studies portrayed societal challenges. The demanding caregiving role does not only interfere with their day-to-day work, but reduce outside social opportunities as well. Taylor and Stanton (2007) reported that social support reduces psychological distress and contributes to physical health and survival. Stigmatisation and social isolation were a prevalent experience among the family caregivers who had to contend with social relationships that were strained by lack of knowledge and negative attitudes about mental illness (Shamsaei et al. 2013). The themes show a consistent link between helplessness, fear, stigma and societal needs.

The review also reported that family caregivers’ inability to effectively cope with caregiving could lead to emotional distress. However, the family caregivers were also able to identify positive aspects of caregiving, especially after being empowered and after being able to support their relative. The use of respite care, where mentally ill relatives were admitted to the hospital to alleviate the effects of caregiving, led to caregivers being relieved from caring for their relative (Salin, Kaunonen & Åstedt-Kurki 2009).

Based on the findings of the meta-synthesis, implications for facilitating the mental health of family caregivers are presented. As mentioned, caregivers got little help in managing the emotional demands of caregiving. A study by Reinhard et al. (2008) revealed that healthcare professionals do not prepare the family for emotional challenges. The findings were relevant to service improvement in terms of mental health supportive care and to facilitate effective coping of family caregivers. One suggestion is that the psychiatric nurses should discuss the role shift, assertive communication and positive emotional coping with caregivers to enable better coping. The caregiver should also be provided with emotional support.

The last recommendation for practice is related to the importance of positive perspectives of caregiving. Being positive helps one to overcome the negative impact of caregiving. Psychiatric nurses should make the caregiver aware of the importance of being positive and help teach them how to develop positive perspectives. By being positive, family caregivers will cope better, which will also improve their mental well-being.

Lastly, it is important for future researchers to be aware of the mental effect of caregiving, to develop appropriate coping strategies for the caregivers. Research should be done on the effect of caregiving on mental health, and programmes that promote effective coping among family caregivers should be developed.

### Limitations

The limitations of the study are that the study was limited to English research studies. This does not mean that people who speak different languages are exempted from caregiving challenges, as is the case in other non-English-speaking countries in sub-Saharan Africa.

Based on the study findings, caregivers bear all the responsibility in caring for their mentally ill relatives.

The results suggest that caregivers were not able to care and have control over their relative living with mental illness; this might instil a sense of guilt. This was, however, not covered in the study and might need further exploration. Differences in caregiving experienced across cultural and ethnic groups have been documented elsewhere (Chakrabarti 2013). More research exploring the role that culture plays on the family caregivers’ experience (given that this study explored caregivers’ experiences in the African context) seems warranted as this was not covered in the study.

## Conclusion

In conclusion, the challenges of coping with caregiving could affect the caregivers’ mental health. The research adds to knowledge about ineffective coping of family caregivers. The study found that the family caregivers were frustrated and experienced a lack of physical and emotional support. The findings also gave insight into the mental aspect of family caregivers. Understanding the caregivers’ experiences could better inform psychiatric nurses on how to assist family caregivers to cope with caregiving. Although limited by a small sample size and confinement to English journals, the research provided insight into the mental aspects of caregiving. The clinical implication of this review emphasised the role of psychiatric nurses in ensuring effective coping for family caregivers through ongoing training and other forms of support.
